# The Antibody Registry: ten years of registering antibodies

**DOI:** 10.1093/nar/gkac927

**Published:** 2022-11-12

**Authors:** Anita Bandrowski, Mason Pairish, Peter Eckmann, Jeffrey Grethe, Maryann E Martone

**Affiliations:** Department of Neuroscience, UCSD, San Diego, CA 92093, USA; SciCrunch Inc, San Diego, CA 92093, USA; SciCrunch Inc, San Diego, CA 92093, USA; Department of Neuroscience, UCSD, San Diego, CA 92093, USA; SciCrunch Inc, San Diego, CA 92093, USA; Department of Neuroscience, UCSD, San Diego, CA 92093, USA; SciCrunch Inc, San Diego, CA 92093, USA; Department of Neuroscience, UCSD, San Diego, CA 92093, USA; SciCrunch Inc, San Diego, CA 92093, USA

## Abstract

Antibodies are ubiquitous key biological research resources yet are tricky to use as they are prone to performance issues and represent a major source of variability across studies. Understanding what antibody was used in a published study is therefore necessary to repeat and/or interpret a given study. However, antibody reagents are still frequently not cited with sufficient detail to determine which antibody was used in experiments. The Antibody Registry is a public, open database that enables citation of antibodies by providing a persistent record for any antibody-based reagent used in a publication. The registry is the authority for antibody Research Resource Identifiers, or RRIDs, which are requested or required by hundreds of journals seeking to improve the citation of these key resources. The registry is the most comprehensive listing of persistently identified antibody reagents used in the scientific literature. Data contributors span individual authors who use antibodies to antibody companies, which provide their entire catalogs including discontinued items. Unlike many commercial antibody listing sites which tend to remove reagents no longer sold, registry records persist, providing an interface between a fast-moving commercial marketplace and the static scientific literature. The Antibody Registry (RRID:SCR_006397) https://antibodyregistry.org.

## INTRODUCTION

Antibodies are key biological resources according to the National Institutes of Health (1), meaning that they are tricky reagents whose performance can vary substantially between laboratories and applications. These reagents have gained substantial notoriety as important culprits in the reproducibility crisis, even entering the popular press when antibody tests failed early in the COVID pandemic (2,3). However, antibodies and antibody-like molecules have also unlocked an entirely new set of medicines, known as biologics, for cancers and many other diseases (4,5). Antibodies are also some of the most ubiquitous laboratory reagents (6), yet in published studies, about half of researchers do not provide enough information to uniquely identify antibodies used in a study (7).

The Antibody Registry, launched in 2010, is a comprehensive on-line catalog of antibodies used in biomedical research. The registry was created under the Neuroscience Information Framework federal contract to UCSD and five other major universities, which also created the SciCrunch infrastructure and launched SciCrunch Inc., the current caretaker of the Antibody Registry. The Antibody Registry was initially created to document how many reagents were available to researchers, a task that was also undertaken by several journals in recognition of the need to carefully identify and validate antibodies using in published studies. In 2011, the Antibody Registry joined forces with the *Journal of Comparative Neurology*, agreeing to cross-list all antibodies in their respective databases (8). In 2014, all antibodies identified in the journal were listed by antibody RRID. The journal *Endocrinology*, which was also planning on creating a very similar reagent database, joined forces with the Antibody Registry in 2015 by opting to ask authors for antibody RRIDs as opposed to keeping a database of antibodies.

The issue of antibody identification in the biomedical literature represented the larger problem of identification of research resources in the methods sections of papers. Not only antibodies, but software tools, cell lines and model organisms suffered from the same lack of identifiability. To solve the broader resource identification problem the Neuroscience Information Framework, Wiley, and the International Neuroinformatics Coordinating Facility (INCF.org), a standards organization for global neuroscience along with program officers from the National Institutes of Health, sponsored a a series of three meetings in 2012/2013 for journal editors and publishers on the issue of increasing identifiability and trackability of research resources in the biomedical literature (https://www.rrids.org/older-history). From these convenings, the RRID pilot project was launched via a working group in FORCE11, which provided the platform to bring together informaticians, publishers and research scientists. The goal of the pilot was to determine how feasible asking authors to provide unique identifiers for certain research resources was, and if such identifiers could improve the identification of reagents like antibodies in participating journals. A portal was created by dkNET that provided a common index for all types of research resources, through which these identifiers could be obtained. These databases were maintained by different authorities, and represented significant previous investments into biomedical infrastructure. To be considered an authority for issuing RRIDs, a registry had to be stable, a recognized community resource, comprehensive (i.e. contain the majority of all possible resources), and allow the addition of new resources. The Antibody Registry was chosen as the authority on antibodies. Within a year we found that antibody identifiability was markedly improved by asking authors for RRIDs (9) and instead of discontinuing the project, the original journals continued to ask authors for RRIDs, and more journals joined over time, impacting a broad swath of the scientific literature. In the following text, we describe the Antibody Registry as a database, the curation process, and the impact that this registry has had on biomedical publishing.

## MATERIALS AND METHODS

### Structure of database

**Figure 1.**
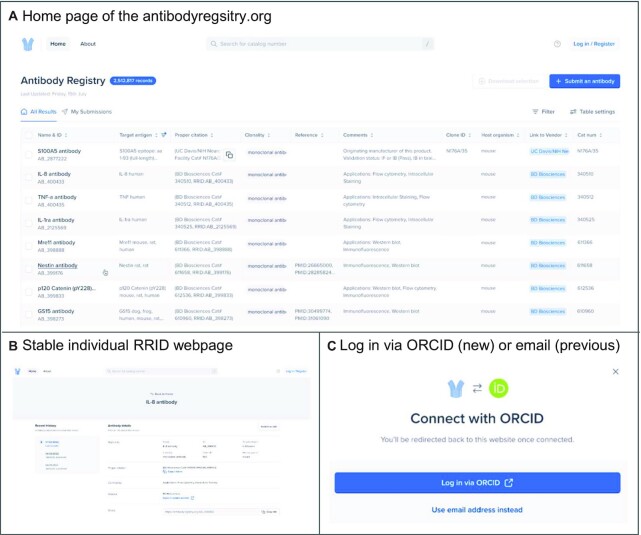
Antibody Registry 2022 redesign of the user interface. (**A**) shows the home page which allows users to search for antibodies (we suggest search for catalog numbers as that is usually the most efficient). Users can now copy the proper citation with a single click available when hovering over the proper citation column. Users can also get more information about the RRID by clicking on the left most column, which is linked to stable webpages shown in B. (**B**) stable webpage for a particular RRID record. These pages display multiple records and the history of this record. (**C**) shows the new login feature through ORCID. Existing registry users are able to use their current accounts.

The Antibody Registry was launched as a product of the Neuroscience Information Framework on top of an ORACLE database in 2009 hosted by UCSD, with direct query from a user interface. The first data came from the UC Davis NeuroMab project, and later from several commercial sources. We updated the registry in 2014 to a MySQL database co-located within the SciCrunch.org Amazon Web Services cluster. This server was the back-end of the registry from 2014 to 2022. Many of the original records added before 2014 now have the March 28, 2014 date, when the final import from ORACLE took place. From 2014 to 2022, a search function was provided by a SOLR index created based on weekly data ‘dumps’ from the database, built via the DISCO system (10). SOLR enabled very fast search, but submitters had to wait 8 days to see their records in the public portal. The 2022 version of the database is running on PostgreSQL v.15 and the registry is again a direct query to a local index of the database, now co-located on the same machine (new user interface shown in Figure 1). This interface will allow simple and more complicated queries such as those against a specific target antigen (filter in target column) suitable for an application (filter by application like WB). Sorting of information will also be available for any column. The Antibody Registry is now hosted at Google Cloud, and the code for the website is under the Apache 2.0 open-source license at a public github repository.

### Funding model

The intellectual property and registry ownership was transferred from UCSD to SciCrunch Inc. via an NIH Small Business Technology Transfer grant, which began to collect membership fees from antibody companies to support and maintain the infrastructure, while keeping submission free to individual antibody submitters. Identifiers issued by the Antibody Registry are made available under a CC0 license. Ownership of the registry with revenue streams will be transferred to a nonprofit, RRIDs.org in the next 3 years, as per stipulation of the current funding from the National Institutes of Health.

### Data ingest

**Figure 2.**
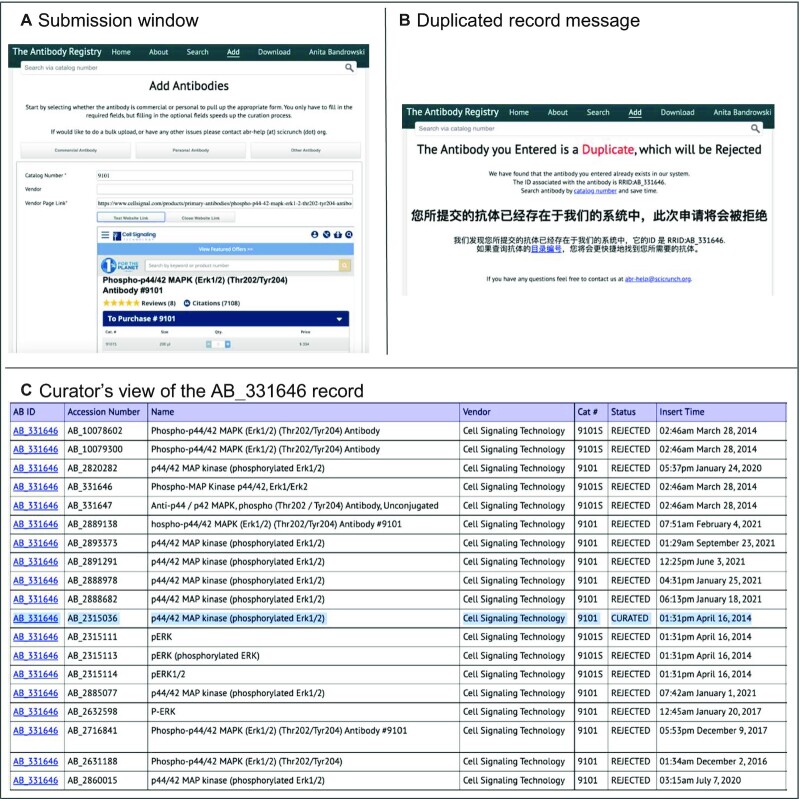
Process of entering a new antibody record in the Antibody Registry. (**A**) shows the submission window. The authors must enter the catalog number and direct URL to the product to obtain an RRID. The URL can be opened in the entry window for easy verification that this is the appropriate record. (**B**) a message to the author when submitting includes either a new RRID or the displayed duplicate message. In both cases, authors are provided an RRID (temporary in the case of a new entry or corrected in the case of a duplicate entry). We updated the duplicate message to also display in Chinese because we noticed that Chinese authors were submitting the same antibody multiple times so we guessed that they were not understanding the message. (**C**) Curator view of 19 records for the same antibody entered by different users over 7 years. One record, highlighted in blue, is curated and available through the public Antibody Registry, while others are only available to curators and submitters.

Data are submitted by individual users and companies or projects using either the user interface (login required) or a spreadsheet that is handled by curation. Submitters must fill in at minimum the catalog number and the direct product URL to obtain a provisional RRID, although additional metadata is encouraged. Submitters receive a provisional RRID via email and a set of instructions about what will happen next (see Figure 2 for screenshots). The curator responds to user requests for a new RRID in one business day (note US holidays are excluded). The curator may confirm the provisional RRID, get back to the user with record updates that impact the RRID provided by the registry, or in rare cases, the curator must wait for a response. Antibody companies prepare their product listings, typically their full catalogs, and send them to the Antibody Registry curators, who examine the files using a set of custom scripts that check data integrity, and then upload the data to the registry database. Curators return the file with RRIDs to companies, who can then add these identifiers to their websites. Several antibody companies, including the Developmental Studies Hybridoma Bank, DSHB, generally submit data before listing products on their website, so each product has an RRID.

### Interacting with data

Data can be viewed and interacted with in several ways. The antibodyregistry.org website allows users to browse, filter and search the catalog of antibodies by catalog number, metadata such as vendor, target or other visible information, but we recommend to users specifically looking for an RRID that they use catalog numbers to search for relevant information. This suggestion is important because authors who search for antibody targets and company names are often dismayed at the number of antibodies that are available. This confusion often generates questions to curation, which are solved by simply looking up the catalog number in laboratory records and searching using that information. Computational users are able to download the full database as a comma-separated values (.csv) file (a total of four fields are under a CC0 license and are available in the file after logging in), or using the SciCrunch.org → API to access a queryable index of the data. The field descriptions are included in the supplemental file to this manuscript.

### Statistics

Tables and figures presented in this manuscript were obtained by querying the Antibody Registry database unless stated otherwise. Descriptive statistics such as counts and means were obtained in Microsoft Excel (RRID:SCR_016137). To obtain the number of individually submitted antibodies, we exported the relevant fields from the database, and found the timestamp of records. We did not summarily remove all curator created records, because we frequently submit records for users that were sent through email, although such requests tend to consist of fewer than five antibodies. When >20 records were included by a single user in a 24 h timeframe, it was considered a bulk action and was removed from Table 1, unless the timestamps were greater than thirty seconds apart (bulk operations consist of records that are entered <1 s apart).

**Table 1.** Submission statistics for individual users 2020–2022

	2020	2021	2022*
Count of submitted antibody records	2890	3041	2024
* Curated antibody records	2258	2394	1444
* Rejected antibody records	632	647	576

Commercial antibodies	2594	2797	1911
Non-profit antibodies	60	24	22
Personal antibodies	234	201	85

Unique users	915	967	624
Vendors	327	329	253

Weekly average	55	56	72

### Coverage of the Antibody Registry

**Figure 3.**
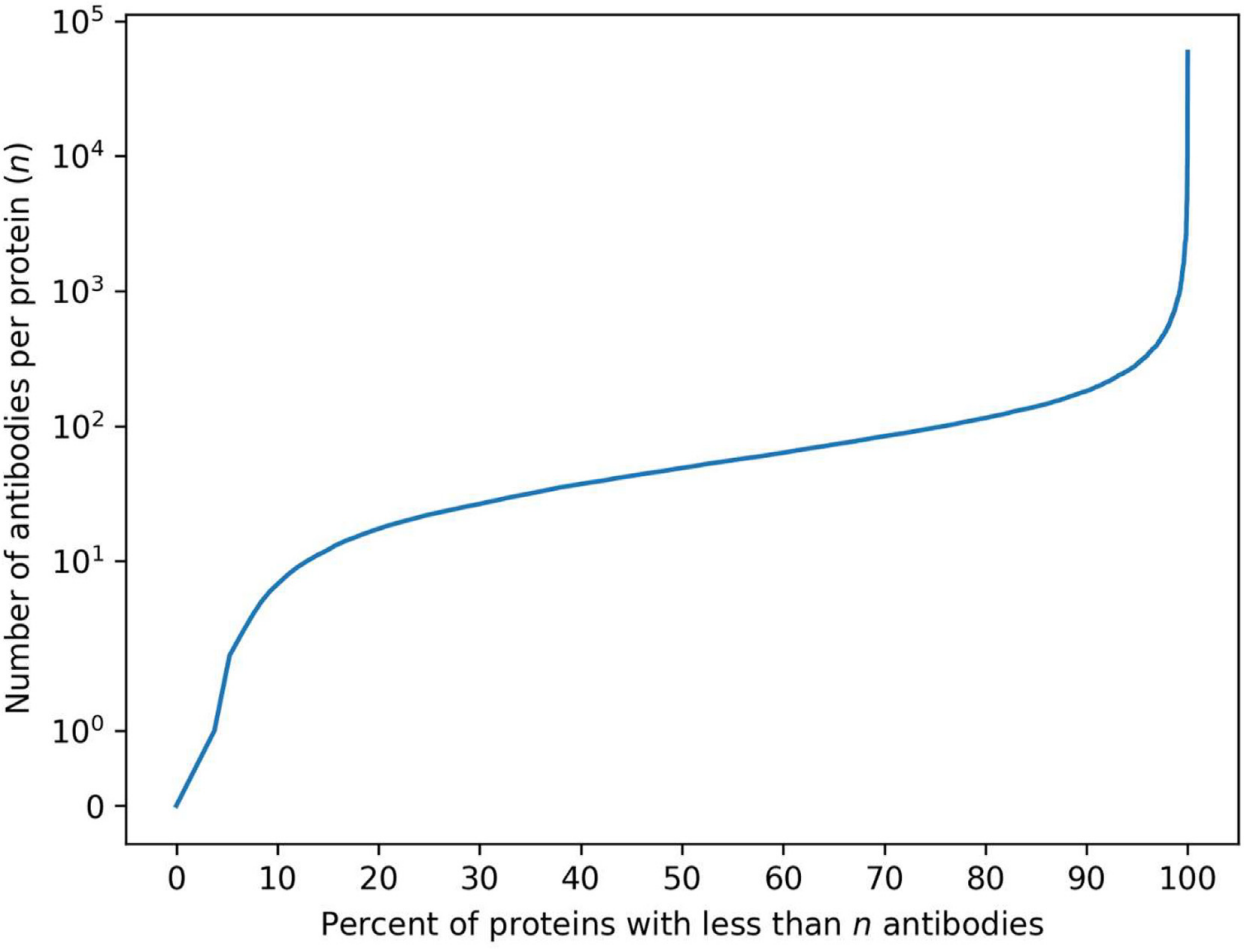
Percentage of the proteome that is covered by antibodies. This cumulative plot shows that about 10% of the total proteins have less than 15 antibodies, about 10% of proteins have more than 100 antibodies, but roughly 80% of known human proteins have 15 to about 100 antibody reagents available for researchers. While we do not know if there are high quality antibodies validated for each target, we do at least know that most of the proteome is accessible by these key reagents. For a listing of protein names and the associated counts please see the supplementary data file.

For Figure 3, the list of human proteins was obtained from UniProt (RRID:SCR_002380), where the protein (a) had evidence of its existence at the protein level, (b) was reviewed and (c) was a human protein. Following these requirements, the following query was constructed: https://www.uniprot.org/uniprotkb?query=(existence:1)%20AND%20(reviewed:true)%20AND%20(organism_id:9606), which was run on 12 May 2020. For each protein name (only taking the first name if there are synonyms), the Antibody Registry index (available at https://scicrunch.org/resources/data/source/nif-0000-07730-1/search) was queried for each protein name on 12 August 2022, and the number of results returned for each query was recorded (see supplemental data file). The top 30 genes with the largest number of results were manually inspected to remove cases where the result count did not accurately reflect the true number of antibodies, e.g. the gene ‘CAT’ matching to all entries with ‘Cat#’. For cases where gene names could potentially overlap with other terms, as determined by a human curator, the number of results were replaced with the results of a manual query to the Antibody Registry index where the ‘Target Antigen’ field was required to match the gene name. While this underestimated the true number of antibodies as gene names or synonyms are also found in the antibody name field, it was preferred for such cases where the default query is a significant overestimate.

Information about the use of RRIDs in the scientific literature is gathered by curators weekly using the SciBot semi-automated workflow described previously (11; Section ‘Corpus of papers containing RRIDs’). The data is made freely accessible via the Hypothes.is API (RRID:SCR_000430). To obtain statistics on usage of Antibody Registry RRIDs in the literature, we queried a local version of the Hypothes.is database (last updated on Aug 8, 2022) to return counts of all RRIDs that include ‘RRID:AB_’, but are not marked as duplicates. These data can be viewed in Hypothes.is at https://hypothes.is/users/SciBot, as annotations on the individual papers (https://hyp.is/aVKDGt_PEeu4jjuX5OipXQ/www.ncbi.nlm.nih.gov/pmc/articles/PMC8250850/), on resolver pages as papers associated with a particular antibody record (e.g. https://n2t.net/RRID:AB_2314866), or in a computationally accessible way by adding ‘.json’ as suffix to the RRID (e.g. https://n2t.net/RRID:AB_2314866.json).

## RESULTS

### Overview

The Antibody Registry contains 2 525 197 records that have been curated, 295 331 that have been ‘rejected’ and 6 are in the ‘queue’ as of 09/09/2022 (54 979 records representing a failed bulk upload in 2014 do not have any tag and are only visible to curators). By type, there are 2 765 555 commercial, 6163 personal and 4102 non-profit antibodies. There are 10 747 users who have registered an account (another 18 071 are from accounts detected to be spam and are banned), and currently 121 of these have linked their account to ORCID. Most users (5857) are associated with ‘.com’ email addresses, 2243 with ‘.edu’ and 109 with ‘.gov’; the rest of the users are mostly associated with country-specific addresses (‘.it’, ‘.de’, etc.). An average of 18 users per week submit an average of 55 antibodies (see Table 1), however institutional users and curators can submit or update hundreds of thousands of records as complete product catalogs are imported. Indeed, support for the Antibody Registry comes in part from institutional memberships, including small and large antibody companies listed on the ‘Home’ page under partners. These members generally contribute their entire catalog each quarter or year, so that their records are up to date and links are accurate. Partner companies often list the RRIDs on their webpages (e.g. DSHB, BioLegend, BD), just as chemical companies list the chemical CAS numbers.

### Features of the Antibody Registry

The Antibody Registry has gone through a substantial redesign in 2022 to better serve our user base. The goal of the registry is to encourage users to search for and quickly identify the reagents that they used in their paper. To serve this goal, the 2022 update includes making the search page the main landing page of the Antibody Registry website (see Figure 1 for design). Frequently asked questions, terms of use and other standard pages are available under the question mark icon next to the login option. Users do not need to log in to search for reagents, but need an account to submit data. Because users from some countries are unable to create an account, the 2022 redesign enabled the option to log in via ORCID (Figure 1C). We hope this will help reduce submitter frustration and the burden on curators.

### Identifiers

Each antibody record is based on a unique combination of vendor name and catalog number, and contains a set of metadata including URL, clonality and the name of the antibody (Figure 1B, see supplement for additional detail). Each antibody record that has been approved by curation is available under the following URL syntax pattern https://antibodyregistry.org/AB_90755, a standard practice for databases that issue compact identifiers (12).

#### When a new identifier is issued

Antibody identifiers, RRIDs, can have multiple records (different accession numbers). Conjugated antibodies and antibodies that have different formulations (e.g. supernatant versus pure) are given their own records because these products are expected to work somewhat differently. However, catalog numbers that track different volumes (–50 ul) within a vendor, or vendor catalog number pairs that track the same product from two different companies, are not intended to be given a new record because we expect that buying more or less of a reagent from company A or B should not change the reagent (see more about the practical aspects of implementing this policy in the Discussion section).

#### Vendor names are not straightforward

Vendors are represented by names, identifiers, and URL patterns. We began in 2012 by asking authors to fill in vendor name information (e.g. Millipore), but realized that even with autocomplete this information was quite variable due to lexical variability and companies buying each other (Millipore Inc., Millipore.com, Chemicon, EMD, Sigma etc.) leading to additional curation work. Therefore, we updated our instructions to allow submitters to fill out only the URL for the product and catalog number. URL patterns are recorded for each vendor and are used to automatically look up the vendor name. These patterns are less variable than company names and submitters receive more accurate antibody identifiers (this is most critical when a duplicate submission is made). Furthermore, providing URLs has two advantages: it is easy for submitters, and we can display the company webpage right in the submission form enabling easy verification and copy/pasting of information. According to google analytics it takes submitters an average of 2:33 to complete a submission (Figure 2A).

#### Identifier persistence

The Antibody Registry adheres to recommendations of the FAIR (findable accessible interoperable reusable) data principles ensuring that metadata for a given antibody persists even if the antibody is discontinued. Therefore, the Antibody Registry, unlike many commercial antibody finding websites, does not remove records because the primary purpose of the registry is to function as the interface between the archival scientific literature and a fast-moving reagent market. In the reagent market, many companies rotate stocks, creating or licensing new antibodies and discontinuing others. Conscientious vendors provide information about the discontinuation, but many vendors do not. To provide a stable resource for researchers and publishers, the Antibody Registry lists discontinued antibodies alongside the currently offered antibodies. Partner companies work with us to mark which antibodies are discontinued and those records get a consistent ‘Discontinued on (date)’ tag. As of 9 August 2022 there were 303 280 discontinued antibodies in the registry. Most records for discontinued antibodies come from our partner companies. Antibodies from companies that do not work closely with the Antibody Registry tend to be discontinued without letting us know, therefore we update records one-by-one as we find them. In one case, all polyclonal antibodies from Santa Cruz Biotechnology were discontinued as per action from the USDA (13), thus we were able to add the ‘discontinued’ tag on all records even though the company is not a partner. Curators monitor news channels to determine if companies have merged or if other major events have taken place in the antibody marketplace.

#### Handling duplicate identifiers

As mentioned above, there may be multiple records for a single antibody. In most cases, a record that duplicates another record will be automatically flagged by the database at submission and although the record is created, the submitter is made aware of the duplicate and is provided the correct identifier for the reagent. These records are rejected and never made public. However, not all duplicates are detected automatically or caught by curators. When curators discover that two antibody records are identical, they consolidate the records (11 976 records have been manually consolidated after records were made public). When curators reconcile the records they leave a note in the comment section. Duplicates in bulk operations are detected when records are entered because the vendor and catalog number constitute a set of defining information and new records can be linked to older records, but when records are entered by individual submitters problematic records can be generated and must be consolidated afterwards (e.g. AB_2338713). For one popular antibody, AB_331646, there were 19 records created between 2014 and 2021 for this antibody, all rejected except one (Figure 2C). It is the policy of the Antibody Registry to keep the lowest number (oldest record) as the public identifier, and to reconcile the records with higher accession numbers into this RRID. The RRID resolver will display a valid page for the accepted RRID (e.g. https://scicrunch.org/resolver/RRID:AB_331646) and the accession number of the record (e.g. https://scicrunch.org/resolver/RRID:AB_2315036). In future releases of the RRID resolver we anticipate that all reconciled accession numbers will be available for resolution even when a record is rejected.

### Curation workflow

Antibody records from antibody companies are aligned by custom scripts by the curator and submitted via spreadsheet to the database by the curator. This process accounts for most updates to the antibody registry because the entire catalog of a company can be updated simultaneously. We keep any comments made by curators or individual users and carefully merge those. In 2013, we allowed several companies to add records directly to the Antibody Registry database, but this resulted in significant reduction in record quality as companies tended to insert unallowed characters, and created multiple records for the same products due to undetected changes in catalog numbers. We discontinued the practice within a year and have been working to reduce the duplicates created during this time period.

#### User initiated commercial or non-profit antibody registration

Individual users/authors can submit a new antibody record from any commercial source by filling out as little as two pieces of information, the antibody catalog number and the direct URL to the product page (see Figure 2A). The database generates an entry with an accession number, a ‘temporary RRID’, and provides information about the curation process to the submitter. If the entry is detected to be a duplicate, the author is informed in both English and Chinese. In most cases the curator updates the antibody within a business day of submission and sends an email to the submitter confirming the addition.

#### User initiated personal antibody registration

When a personal or ‘other’ antibody type is submitted, there is significantly more back and forth with the curators. Personal antibodies are typically made by the submitting lab, while ‘other’ antibodies are a catch-all category for reagents that were made for a lab by companies, other laboratories or core facilities. The curator frequently must access and read the manuscript in which the reagent was first described, especially when the submitter and the creator of the antibody are not the same individual. The curator also makes an attempt to reach out to the original creator of the antibody both to confirm the accuracy of the submitted record and to let the original creator know that the record has been created. At times this is not possible because the antibody creator is either no longer in an academic lab or deceased. Authors registering personal antibodies are therefore asked for additional patience and time to ensure that the records are as accurate as possible.

### Genome coverage

Figure 3 shows the percentage of the human genome that is associated with antibody reagents. We queried the Antibody Registry with a set of Uniprot-derived protein names representing 16 281 human proteins. The input list can be found in the supplemental data file, as well as the number of Antibody Registry results that match this list. This analysis should be considered an estimate because we did not analyze each record to determine whether all results were completely accurate. We found that 96.2% of all human protein names returned at least one result, illustrating the broad coverage of the human genome in the Antibody Registry.

### Impact of the antibody registry

The Antibody Registry's identifiers for antibodies, RRIDs, have been used in the scientific literature 343 126 times from 1 February 2014 to 8 August 2022. Antibody RRIDs are required in several journals including *Nature Protocols, Endocrinology* and the *Journal of Comparative Neurology* (8,14). They are encouraged or strongly encouraged in about 1000 journals using various means. The *Frontiers* family of journals asks for RRIDs in a submission checklist for authors, the *Journal of Neuroscience* asks authors in letters to authors, *eLife* helps authors find RRIDs, the *Cell Press* family journals require a Key Resources Table (includes RRIDs) in all papers, while *Nature* and many other journals include RRIDs in their instructions to authors documents. We have studied the effectiveness of these different methods of requesting RRIDs previously (9,15), and found that journals actively requiring antibody RRIDs have over 90% compliance while journals that ask with only passive instructions to authors have about 1% compliance. Letters and checklists are more effective than passive instructions to authors, but author compliance still hovers around 10%. More active engagement such as personal emails from editors can get >50% of authors to comply. We have identified 1041 journals that contain less than six papers with RRIDs (see supplemental data file), constituting about half of the total 2036 journals that have at least one paper with an RRID. These papers are likely to be written either by authors who have adopted RRIDs as a citation practice or have been prepared for a journal that requests RRIDs and were eventually published elsewhere.

**Figure 4.**
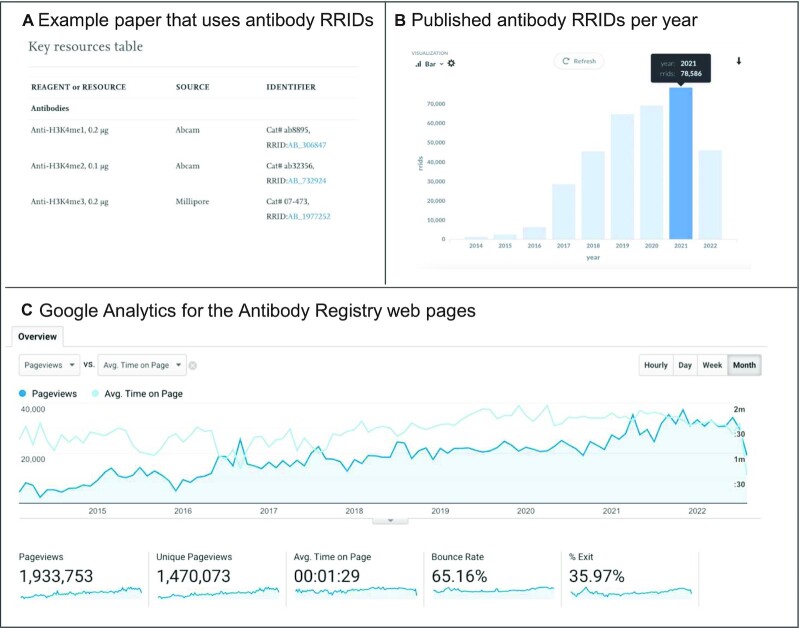
Antibody Registry access statistics. (**A**) shows an example paper (17) that contains RRIDs linked to the Antibody Registry for all RRIDs. (**B**) Counts of antibody RRIDs as of August 8, 2022 captured by curators used in papers such as that shown in (A), per year. (**C**) Monthly access statistics provided by Google analytics for the Antibody Registry from 1 February 2014 until 8 August 2022.

Usage of the Antibody Registry seems linked to the usage of antibody RRIDs in the scientific literature (see Figure 4), and although we can’t know the intent of the users of the Antibody Registry, we do see a rough correlation between increase in site usage and the increase of antibody RRIDs in the literature. We see that in the last several years about four pages are accessed for each antibody RRID in the published literature. If usage of the website precedes the presence of RRIDs in the literature, we should see ∼20% more RRIDs in 2022 over 2021.

## DISCUSSION

### Impact

The Antibody Registry has had a measurable and significant impact on the identifiability of antibody reagents in the scientific literature. Over 300 000 RRIDs for antibodies have been used across 46 500 papers and 2000 journals. In nearly all cases, these antibody references allow readers to find the reagent, and to understand where else the reagent was used. However, we believe that the impact goes much further. While it is still easy to find papers citing antibodies with insufficient information to uniquely identify the reagent, such as authors just citing the protein and vendor, a truly terrible practice, it is getting less common. A recent study of all accessible antibody sentences (in ∼2M documents) in the open access literature found that antibody catalog numbers or RRIDs (making them uniquely identifiable) in papers is becoming much more common, going from 12% of antibody references in 1997 to 31% in 2020 (16). It is unknown to what extent this welcome change is due to efforts of the Antibody Registry, but with over 300K antibody RRIDs in the scientific literature, the contribution is not insignificant.

### Data quality

The Antibody Registry has faced significant data quality issues. We often do not know how many products exist for a given target, we can only report on how many different vendors offer antibodies against a target (15). Some of the data quality issues reflect the quality of information we receive. Commercial catalogs do not always refer to the gene or protein numbers for each reagent, and there may be duplicate records that remain in the database from various uploads or additions of data. Some of the reasons why duplicate records exist in the Registry is because we have uploaded several full catalogs that we later discovered contain the same products (e.g. Chemicon and Millipore) and then merged the records in bulk because they were the same antibody, but we have not yet adjudicated which accession numbers will be preserved. Unfortunately, it has not always been transparent to users why some duplicates exist. In these cases, curation reduces the duplication of antibody records over time as we examine the relationships between companies (currently nearly 12 000 records have been manually consolidated, and many more have been consolidated in bulk operations especially as we bring on additional partners).

We strongly encourage companies to adopt more transparent practices to reduce the duplication of antibody products that is responsible for many lost hours of research effort investigating antibodies that are presumed to be independent, but are in fact the same reagent (6). Tracking duplicate antibodies is difficult in an industry that profits from frequent antibody trading between companies (15). The practice of relabeling product identifiers, including clone numbers, is still far too common, although many of the more established companies have now become much more proactive in reducing or eliminating these practices. For example, the full Human Protein Atlas (HPA) antibody catalog is available via Sigma; however all antibodies are listed transparently by their HPA catalog numbers. Similarly, the Biolegend Covance antibodies are clearly listed as originating from Covance. Thermo Fisher recently made all Bethyl antibodies available, but these reagents are sold under the Thermo Fisher brand with same catalog numbers as the original products so they could be more easily cross linked. Researchers should be aware of relabelling practices and should consult the original product data sheets when they buy multiple reagents for a target to ensure that the reagents are indeed different (6,15). In an egregious example, a company unknowingly acquired the same antibody product that they previously sold to a distributor, but the concentration was diluted in the opaque chain of custody. Once discovered the company quickly discontinued the dilute product, but investigators who bought the antibody still wasted significant effort.

### One step together

The Antibody Registry's first goal is to get all reagents that are sold listed and registered, because there is power in having a complete record even if the record is ‘messy’. Therefore, we have always accepted full product catalogs from companies and records that submitters provide, with scant evidence such as pictures of reagent bottles or stained product inserts as evidence that an antibody exists. However, the registry does not plan on increasing the granularity of the antibody records down to lot numbers from the current base of catalog numbers, because we do not believe that authors are ready to provide this information in journal articles. Additionally, issuing and maintaining identifiers for lot numbers and their availability would be prohibitively resource intensive. We encourage researchers to provide this information in their methods section. However, the *Journal of Comparative Neurology* insisted on both catalog numbers and lot numbers from their authors in addition to validation data for all antibodies. 90% of their authors provided catalog numbers, and about 5% provided lot numbers (C. Saper, personal communication). If authors across the literature at least provide catalog numbers it will improve the findability of antibodies. While this step will not be sufficient to solve the reproducibility crisis, we shouldn’t let the perfect be the enemy of the good. The Antibody Registry and RRID initiative have succeeded in greatly improving the identifiability of antibodies used within the scientific literature for researchers, many of whom have at one point been frustrated trying to track down which antibodies were used in a scientific paper.

## DATA AVAILABILITY

Antibody Registry, RRID:SCR_006397, is an open source project available on GitHub https://github.com/MetaCell/scicrunch-antibody-registry.

The SciBot tool, RRID:SCR_016250, is also an open source project available on GitHub https://github.com/scicrunch/scibot.

Each antibody entry is resolved by several resolver services including:

- The name to thing resolver https://n2t.net/RRID:AB_331646
- Identifiers.org https://identifiers.org/RRID/RRID:AB_331646
- SciCrunch https://scicrunch.org/resolver/RRID:AB_331646

## Supplementary Material

gkac927_Supplemental_File
